# Impact of Barbed Suture Closure on Vaginal Cuff Dehiscence Following Robot-Assisted Total Hysterectomy: A Retrospective Cohort Study

**DOI:** 10.7759/cureus.81672

**Published:** 2025-04-03

**Authors:** Shinichi Togami, Furuzono Nozomi, Yusuke Kobayashi, Mika Fukuda, Mika Mizuno, Shintaro Yanazume, Hiroaki Kobayashi

**Affiliations:** 1 Department of Obstetrics and Gynecology, Faculty of Medicine, Kagoshima University, Kagoshima, JPN

**Keywords:** barbed sutures, colpotomy, minimally invasive surgery, robot-assisted hysterectomy, vaginal cuff dehiscence

## Abstract

Introduction

Vaginal cuff dehiscence (VCD) is a rare but serious complication following hysterectomy, with a higher incidence in minimally invasive surgery (MIS). The choice of suture material and closure technique may influence the risk of VCD. Barbed sutures, such as STRATAFIX^™^ Spiral PDS Plus, enhance tissue approximation and eliminate the need for knot tying, potentially improving wound healing. This study aimed to evaluate the incidence of VCD following robot-assisted total hysterectomy (RAH) with vaginal cuff closure using barbed sutures.

Methods

This retrospective cohort study included patients who underwent RAH at Kagoshima University Hospital between July 2017 and July 2024. Patients who had vaginal cuff closure with barbed sutures were analyzed, while those who underwent supracervical hysterectomy or had vaginal cuff closure with non-barbed sutures were excluded. VCD was assessed through pelvic examinations at one, three, and six months postoperatively, or earlier if clinically indicated. The presence or absence of VCD was determined based on findings within six months after surgery. All procedures were performed by certified gynecologic oncologists and laparoscopic surgeons using either the da Vinci^®^ Xi or hinotori^™^ Surgical Robot System. Colpotomy was performed using monopolar coagulation (35 W), followed by vaginal cuff closure with interrupted 0-Polyglactin 910 sutures at the lateral ends and continuous barbed suture closure.

Results

A total of 313 patients were included, with a median age of 55 years and a median BMI of 28 kg/m². Of these, 270 (86%) had malignant conditions, while 43 (14%) had benign conditions. The median operative time was 201 minutes, and the median blood loss was 20 mL. Retroperitoneal suturing was performed in 224 patients (72%). No cases of VCD were observed.

Conclusions

This study demonstrates that vaginal cuff closure using barbed sutures during RAH is a feasible and safe technique, with no instances of VCD reported within the six-month postoperative period. The standardized surgical approach contributed to consistent outcomes across the cohort. These findings suggest that barbed suture closure may effectively reduce the risk of VCD, particularly in high-volume robotic surgery settings.

## Introduction

Hysterectomy is a commonly performed gynecologic procedure used to treat both benign and malignant conditions. Various surgical approaches are available, including abdominal hysterectomy, laparoscopic hysterectomy (LH), robot-assisted total hysterectomy (RAH), and vaginal hysterectomy. With the increasing adoption of minimally invasive surgery (MIS), LH and RAH have gained prominence due to their advantages, such as reduced postoperative pain, shorter hospital stays, and faster recovery [1,2]. However, the growing prevalence of MIS has been linked to a higher incidence of vaginal cuff dehiscence (VCD), emphasizing the need for effective prevention and management strategies [3-5].

The incidence of VCD varies by surgical approach, with MIS procedures carrying a higher risk compared to open surgery. Studies have reported a VCD incidence of 0.75% following LH, which is greater than the 0.38% observed after abdominal hysterectomy [6]. VCD is believed to result from impaired wound healing at the vaginal cuff and excessive mechanical stress, which can lead to bowel evisceration - a severe complication requiring emergent surgical intervention due to the risk of bowel ischemia and necrosis. Several documented cases of bowel evisceration following VCD highlight its clinical significance.

Multiple risk factors for VCD have been identified, including patient-related factors such as vaginal hematoma, smoking, and chronic constipation, all of which negatively impact wound healing [7,8]. Additionally, postoperative factors, such as early resumption of sexual activity and increased intra-abdominal pressure from coughing or straining during defecation, contribute to VCD occurrence [8]. Surgical factors, including the surgical approach, suture technique, and type of energy device used for colpotomy, further influence VCD risk. However, no consensus exists on the optimal technique to minimize its occurrence.

STRATAFIX^™^ Spiral PDS Plus is a knotless, absorbable barbed suture with a 360° spiral configuration of barbs along its surface and is impregnated with triclosan, an antimicrobial agent. This novel suture design enhances tissue approximation and eliminates the need for knot tying, potentially improving wound healing. This study aimed to evaluate the incidence of VCD following RAH with vaginal cuff closure using STRATAFIX^™^ Spiral PDS Plus, assessing its efficacy and safety in VCD prevention.

## Materials and methods

Study overview

This single-center retrospective cohort study was conducted at Kagoshima University Hospital to evaluate the incidence of VCD following RAH with vaginal cuff closure using barbed sutures.

Ethical considerations

The study was approved by the Institutional Review Board of Kagoshima University Hospital (IRB approval no. 20-K04). Due to its retrospective design and the use of anonymized data extracted from electronic medical records, the requirement for informed consent was waived.

Study criteria

Patients who underwent RAH with vaginal cuff closure using barbed sutures (STRATAFIX™ Spiral PDS Plus) between July 2017 and July 2024 were included in the study. Patients who underwent supracervical hysterectomy or those in whom barbed sutures were not used for vaginal cuff closure were excluded.

Surgical procedure

All surgeries were performed by four certified gynecologic oncologists from the Japan Society of Gynecologic Oncology. Among them, two were board-certified laparoscopic surgeons accredited by the Japan Society of Gynecologic and Obstetric Endoscopy. Additionally, all four surgeons held certifications for the da Vinci® Xi and hinotori™ Surgical Robot System.

**Figure 1:**
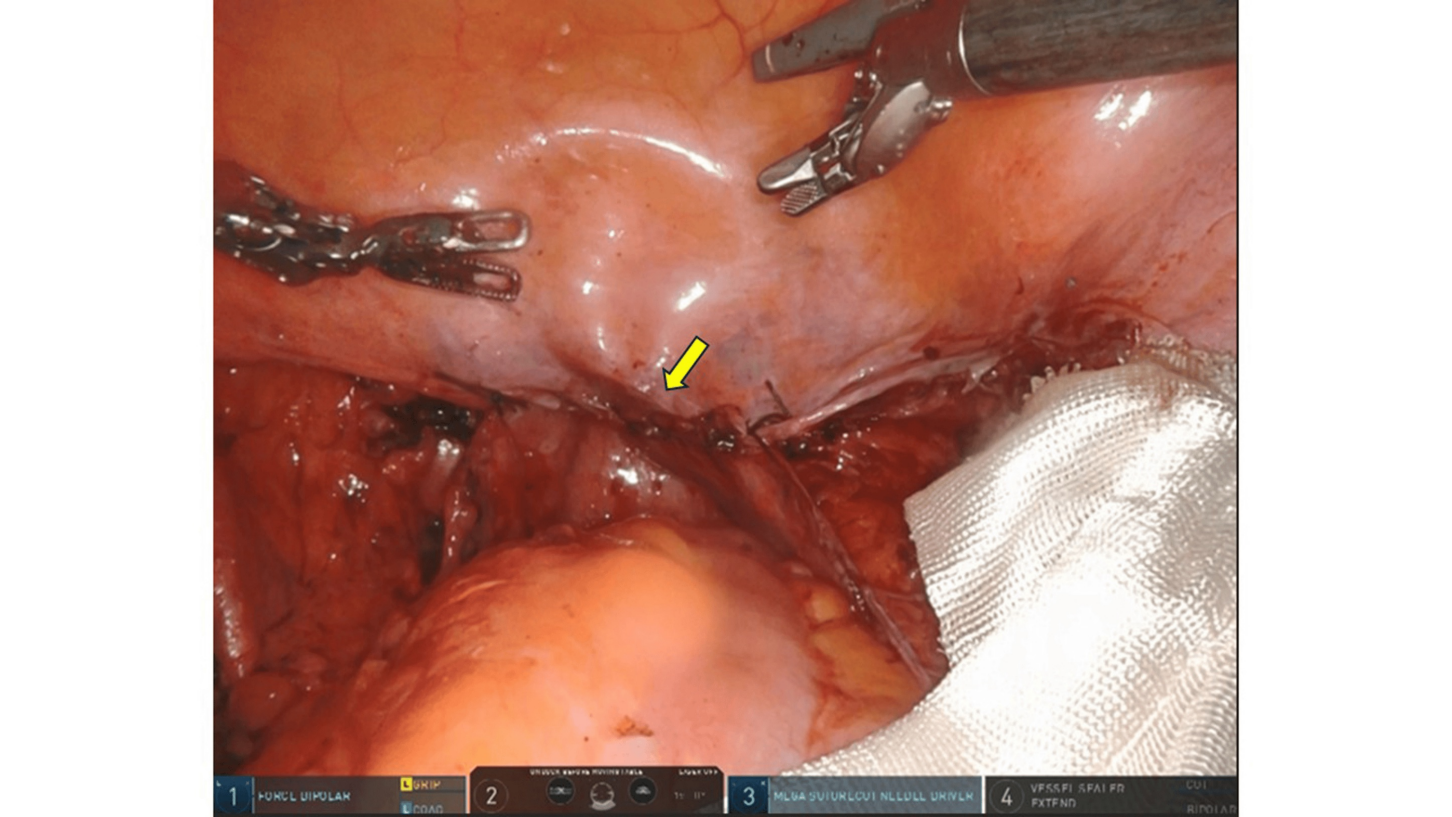
Vaginal cuff closure using a barbed suture with retroperitoneal closure The arrow indicates the area where retroperitoneal suturing was performed following vaginal cuff closure.

**Figure 2:**
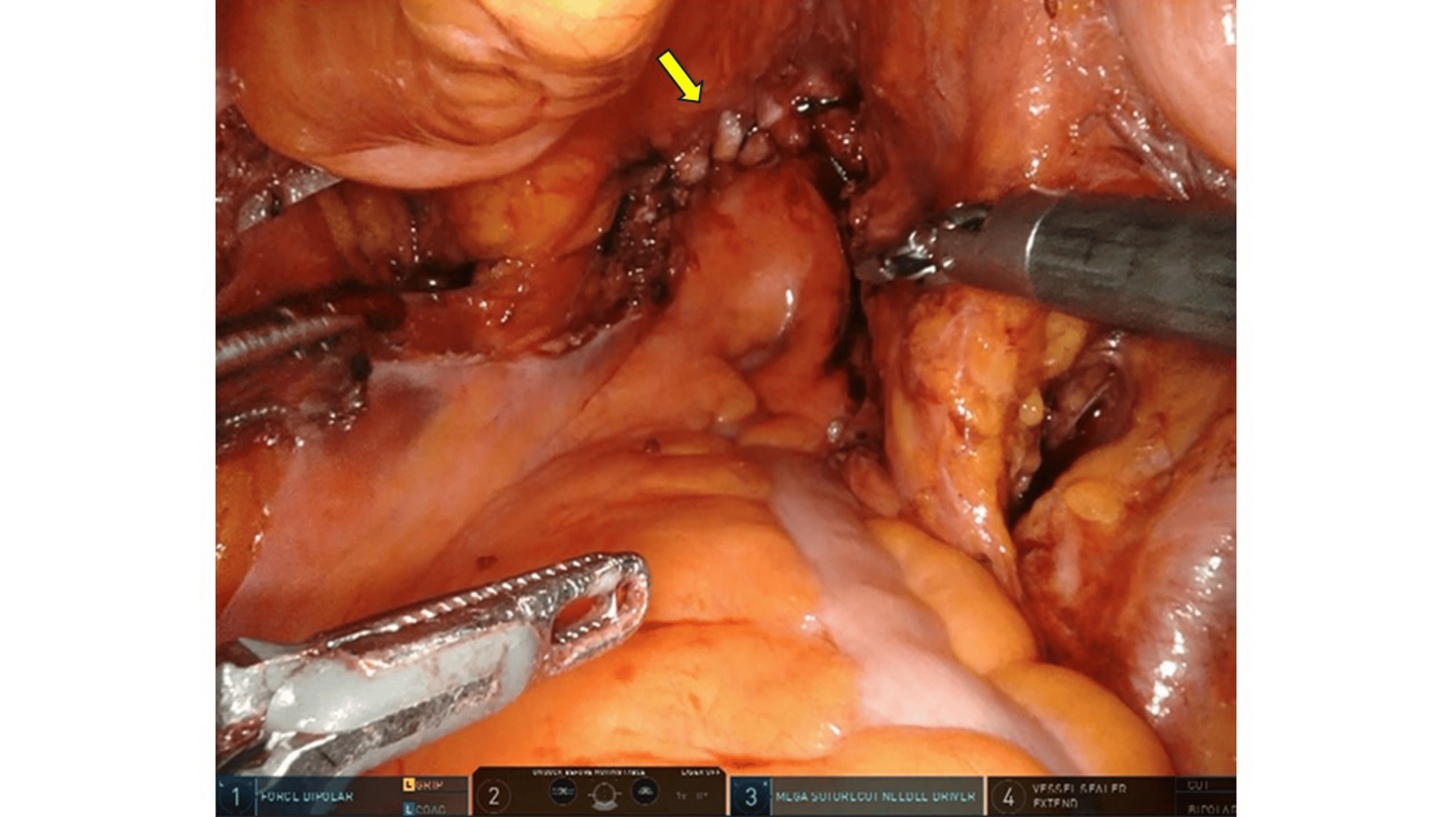
Vaginal cuff closure using a barbed suture without retroperitoneal closure The arrow indicates the site of vaginal cuff closure where retroperitoneal suturing was not performed.

Colpotomy was performed using monopolar scissors in coagulation mode at 35 watts to create a circumferential incision. Vaginal cuff closure began with the placement of interrupted 0-Polyglactin 910 sutures at both lateral ends, followed by a continuous barbed suture using STRATAFIX™ Spiral PDS Plus. Retroperitoneal closure was performed at the surgeon’s discretion (Figure 1, Figure 2).

Assessments

The presence or absence of VCD was assessed through routine pelvic examinations performed at one, three, and six months postoperatively, or earlier if clinically indicated. If a patient underwent a pelvic examination for other reasons within six months after surgery, those findings were also reviewed. If no VCD was detected during the six-month postoperative period, the patient was considered VCD-negative for the purpose of this study. All examinations were conducted by attending gynecologists and documented in the electronic medical records.

Sample size calculation

Since this study aimed to determine the incidence of a rare event (VCD), all eligible patients during the seven-year study period were included. A formal sample size calculation was not performed.

Statistical analysis

Descriptive statistics were used to summarize clinical characteristics and outcomes. As no cases of VCD were observed, a one-sided 97.5% CI for a zero-event proportion was calculated, resulting in an upper limit of 1.15%. This estimation provides a statistical boundary for the true incidence of VCD in this population.

All statistical analyses were performed using JMP version 14 (SAS Institute Inc., Cary, NC, USA).

## Results

A total of 313 patients who underwent RAH were analyzed to assess the incidence of VCD following barbed suture closure (Table 1).

**Table 1:** Clinicopathological characteristics in robot-assisted surgery cases VCD, vaginal cuff dehiscence

Characteristics	Patients (n = 313)
Median age (years)	55 (28-86)
Median BMI (kg/m²)	28 (17.3-53.1)
Surgery indication	
Benign disease	43 (14%)
Malignant disease	270 (86%)
Median operation time (min)	201 (range: 68-638)
Median cockpit/console time (min)	148 (range: 53-520)
Median blood loss (mL)	20 (range: 5-685)
Median length of hospital stay (days)	6 (range: 3-22)
Robotic type	
da Vinci^®^ Xi	268 (86%)
hinotori^™^ Surgical Robot System	45 (14%)
Retroperitoneal suture	
No	89 (28%)
Yes	224 (72%)
VCD	
No	313 (100%)
Yes	0

The median age of the patients was 55 years (range: 28-86 years), and the median BMI was 28 kg/m² (range: 17.3-53.1 kg/m²). Surgical indications included malignant tumors in 270 patients (86%) and benign conditions in 43 patients (14%).

The median operation time was 201 minutes (range: 68-638 minutes), with a median console/cockpit time of 148 minutes (range: 53-520 minutes). The median estimated blood loss was 20 mL (range: 5-685 mL), and the median length of hospital stay was six days (range: 3-22 days).

Two robotic platforms were used in this study: the da Vinci^®^ Xi system in 268 patients (86%) and the hinotori^™^ Surgical Robot System in 45 patients (14%). Retroperitoneal suturing was performed in 224 patients (72%). No cases of VCD were identified within the six-month postoperative period among the 313 patients. The calculated one-sided 97.5% CI for a zero-event proportion was 0-1.15%, indicating that the true incidence of VCD in this cohort is likely below 1.15%.

## Discussion

This study investigated the incidence of VCD within six months postoperatively in patients who underwent RAH with vaginal cuff closure using barbed sutures. Among the 313 patients, no cases of VCD were observed during the defined follow-up period, regardless of age, BMI, or retroperitoneal suturing status.

The reported incidence of VCD following MIS varies, with rates ranging from 0.32% to 1.0% in LH and from 0.22% to 1.49% in RAH [9-14]. However, in our study, VCD did not occur in any of the 313 patients. Among the surgical risk factors for VCD in MIS, narrow suture width resulting from magnified visualization has been proposed as a potential contributor. Fuchs Weizman et al. [8] compared interrupted and continuous suturing techniques and concluded that continuous suturing reduced the risk of VCD. Similarly, Uccella et al. [15] reported that barbed sutures were associated with a lower risk of VCD compared with non-barbed closure. In contrast, Cannone et al. [16] found no significant difference in VCD risk between Vicryl and PDS sutures. These findings suggest that barbed suture closure during RAH may contribute to a lower incidence of VCD. The six-month follow-up period was chosen based on previous studies indicating that the majority of VCD events occur within the first three to six months after surgery [12,15]. Therefore, this time frame was deemed appropriate for capturing clinically relevant VCD events while ensuring consistency in outcome assessment.

Few studies have examined the relationship between VCD and retroperitoneal suturing. Hada et al. [9] evaluated 677 patients with LH and identified VCD in four patients (0.6%), but found no statistical correlation between retroperitoneal suturing and VCD. Similarly, our study found no cases of VCD in the 89 patients (28%) without retroperitoneal suturing, supporting the findings of Hada et al. [9].

Another debated factor in MIS-associated VCD is the use of energy devices for colpotomy. Previous studies have investigated whether energy-based colpotomy increases the risk of vaginal cuff separation. Taşkın et al. [11] compared monopolar coagulation and cut mode and found no significant difference in VCD incidence. Similarly, another study found no significant difference in VCD occurrence when colpotomy was performed using a cold scalpel, advanced bipolar devices, monopolar electrosurgery, or a harmonic scalpel [8]. In our study, monopolar colpotomy was performed in all patients, with no VCD observed. This suggests that monopolar energy use may not be a significant risk factor for VCD.

This study has notable strengths. All patients underwent a uniform surgical procedure - RAH with barbed suture closure - performed by experienced surgeons at a single center. This consistency reduces variability and strengthens the internal validity of the findings. The relatively large cohort over a seven-year period also enables a reliable estimation of VCD incidence, a rare postoperative complication. A retrospective design was chosen because VCD is rare, and data were readily available from electronic medical records. A prospective study would have been time-consuming and less practical for this objective.

However, limitations include the potential underreporting of asymptomatic VCD due to reliance on chart documentation. Additionally, the absence of a control group limits direct comparison with other suture methods, and the single-center setting may affect the generalizability of the findings. Despite these limitations, the absence of VCD in this large, homogeneous cohort supports the safety and feasibility of barbed suture closure in RAH.

## Conclusions

In this retrospective cohort study, no cases of VCD were observed within the six-month postoperative period among 313 patients who underwent RAH with barbed suture closure. Given that previous studies have shown that most VCD events occur within three to six months after surgery, this time frame was considered clinically appropriate for capturing relevant outcomes. The findings suggest that barbed suture closure may be a safe and effective technique for preventing VCD in robotic hysterectomy, irrespective of patient age, BMI, or retroperitoneal suturing. While the study is limited by its retrospective design and the absence of a comparison group, the use of a standardized surgical approach and consistent follow-up enhances the reliability of the results. Further prospective studies with longer-term follow-up are needed to confirm these findings and assess long-term safety.
